# Supplementary material for: Acute depletion of BRG1 reveals its primary function as an activator of transcription
Source: Nat Commun. 2024 May 29;15:4561. doi: 10.1038/s41467-024-48911-z (PMC11137027; doi:10.1038/s41467-024-48911-z)
Supplement: Supplementary file 1 — Supplementary Information [file 41467_2024_48911_MOESM1_ESM.pdf]

Supplementary Information File

Acute depletion of BRG1 reveals its primary function as an activator of transcription

Gang Ren[1,2,6], Wai Lim Ku[1,6], Guangzhe Ge[1,6], Jackson A. Hoffman[3], Jee Youn Kang[1], Qingsong Tang[1], Kairong Cui[1], Yong He[4], Yukun Guan[4], Bin Gao[4], Chengyu Liu[5], Trevor K. Archer[3], Keji Zhao[1,#]

[1] Systems Biology Center, National Heart, Lung, and Blood Institute, NIH, Bethesda, MD 20892, USA.

[2] College of Animal Science and Technology, Northwest Agriculture and Forest University, Yangling, Shaanxi 712100, China;

[3] Epigenetics and Stem Cell Biology Laboratory, National Institute of Environmental Health Sciences, NIH, Research Triangle Park, North Carolina, 27709, USA.

[4] Laboratory of Liver Diseases, National Institute on Alcohol Abuse and Alcoholism, National Institutes of Health, Bethesda, MD. 20892, USA.

[5] Transgenic Core Facility, National Heart, Lung, and Blood Institute, NIH, Bethesda, MD 20892, USA.

[6] These authors contributed equally to this work.

[#]Correspondence: zhaok@nhlbi.nih.gov.

This pdf file includes:

- Supplementary Figure 1-13
- Supplementary Table 1
- Supplementary Reference

Supplementary Figure

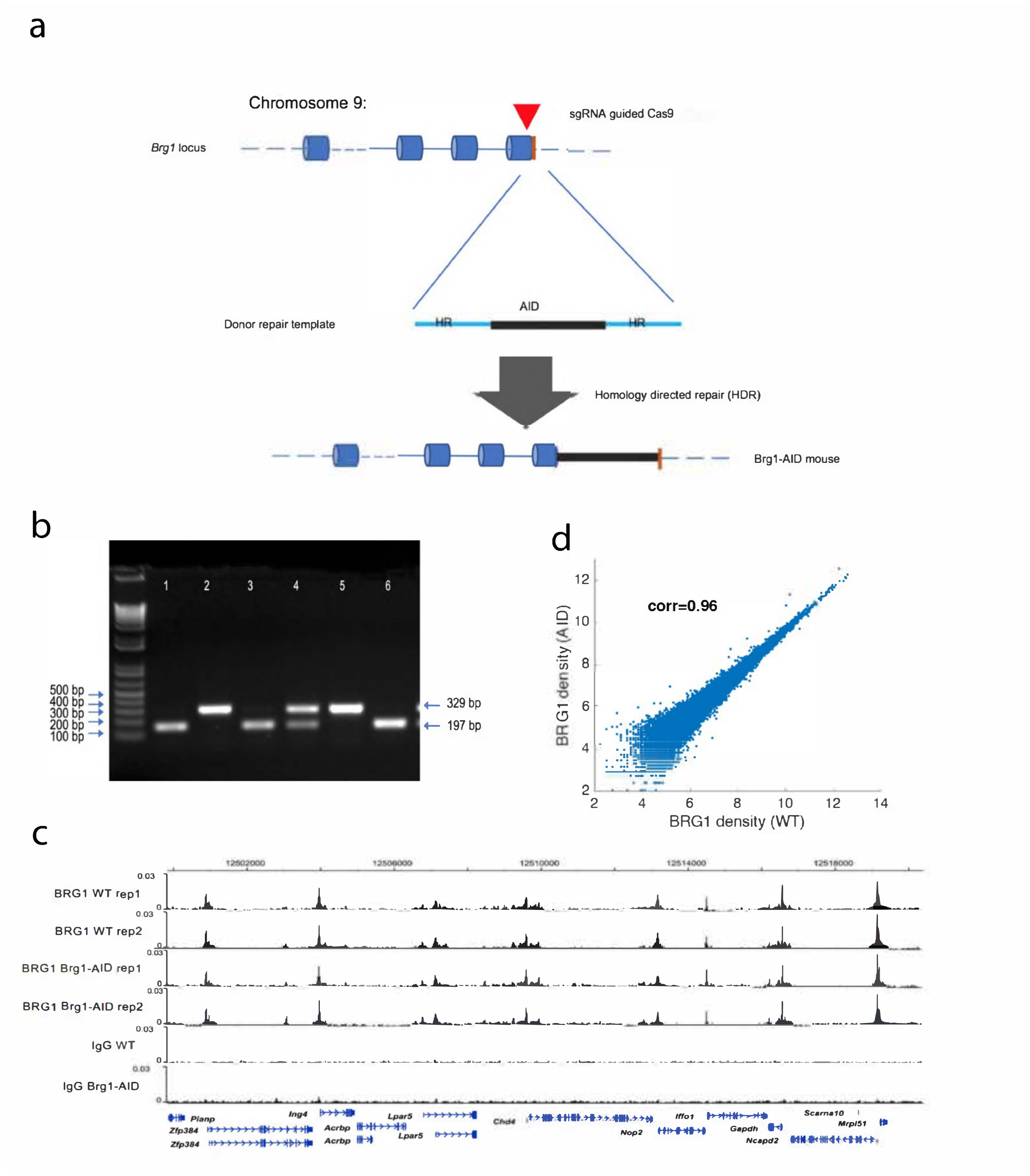

Supplementary Figure 1. Tagging BRG1 with AID does not change its binding profiles on chromatin.

a. Scheme for generating the *Brg1-AID* fusion gene in mice using CRISPR/Cas9-mediated knock-in strategy. AID (132 bp) DNA fragment was inserted before the *Brg1* gene stop codon by CRISPR/Cas9 mediated Homology directed repair (HDR).
b. Genotyping of *Brg1-AID* knock-in mice. The agarose gel image showing the different DNA fragments amplified from the AID sequence knock-in mice (329 bp) and wild type (WT) mice (197 bp). Lanes 1, 3, 6 indicate wild type mice; Lane 4 indicates heterozygous knock in mice; and lanes 2, 5 indicate homozygous knock in mice.
c. Genome browser snapshot showing the BRG1 ChIC-seq signals in wild type T cells (top tracks 1, 2) and *Brg1-AID* knock-in T cells (track 3, 4), and IgG control signals (track 5, 6) in these cells.
d, The genome-wide binding densities of BRG1 in wild type T cells and BRG1-AID in *Brg1-AID* knock-in T cells measured by ChIC-seq are highly correlated.

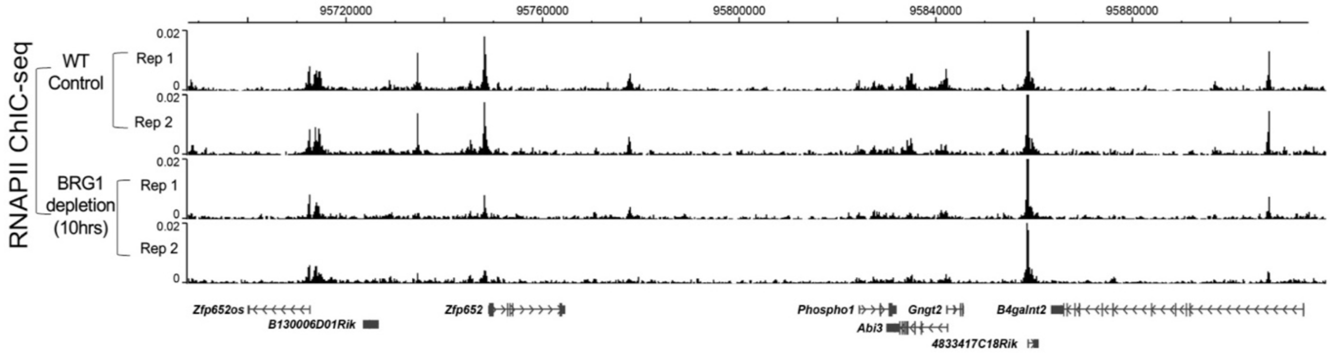

Supplementary Figure 2. Acute depletion of BRG1 results in decreased binding of RNA Pol II on chromatin. CD4$^+$ T cells isolated from *Brg1-AID* mice or wild type mice were transduced with osTir1 expression retroviral particles for 24 hours and treated with auxin for 10 hours. The cells were then subjected to ChIC-seq analysis with Pol II antibodies. The genome browser snapshot shows the RNA polymerase II binding profiles in wild type T cells (top tracks 1, 2) and BRG1-depleted T cells (bottom tracks 3, 4).

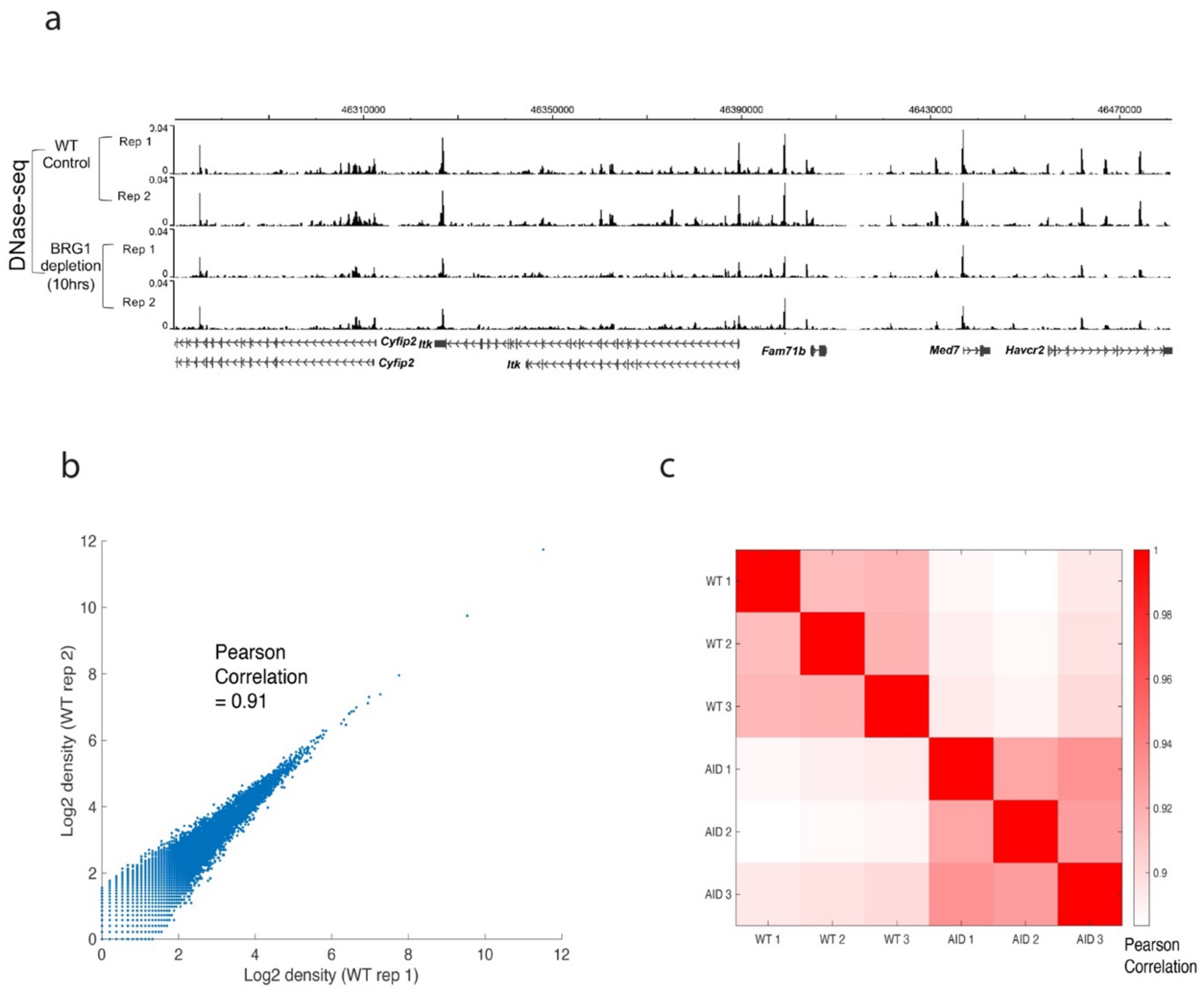

Supplementary Figure 3. Acute depletion of BRG1 results in decreased chromatin accessibility at transcriptional regulatory regions.

a. The cells were treated as described in Supplementary Figure 2 and subjected to DNase-seq analysis. The genome browser snapshot shows the DNase-seq signals in wild type T cells (top tracks 1, 2) and BRG1-depleted T cells (bottom tracks 3, 4).

b. Scatter plot showing the reproducibility between two replicates of DNase-seq data in WT cells. The Pearson correlation is 0.91.

c. Heatmap showing the Pearson correlation among replicates of DNase-seq data in WT and AID cells.

a

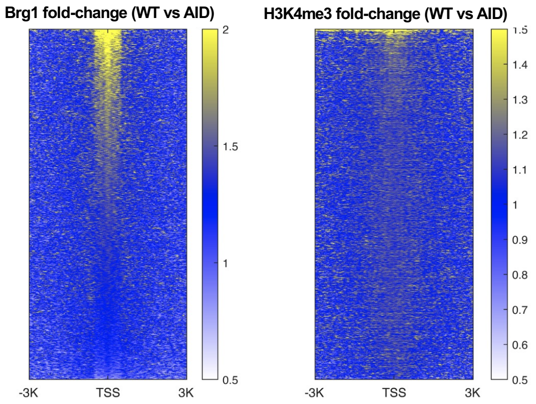

b

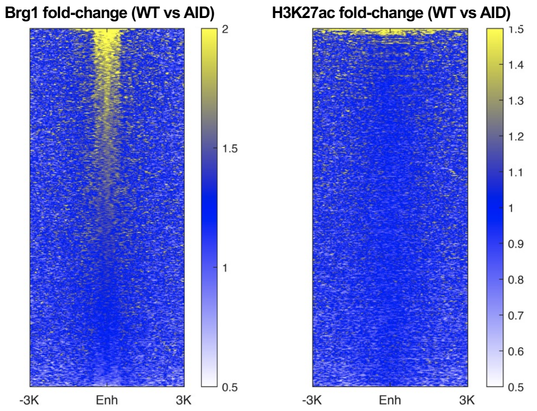

Supplementary Figure 4. Comparisons of changes in BRG1 bindings and Histone modification signals upon BRG1 depletion by auxin treatment in T cells.

a. The left panel displays heatmaps depicting the BRG1 fold-change between WT control and AID-mediated BRG1-depleted cells. The right panel illustrates the H3K4me3 fold-change between WT and BRG1-depleted cells. These heatmaps are centered around TSS regions that co-localized with BRG1 peaks in either WT or AID CD4+ T cells. In the color scale, higher fold-change values greater than 1 indicates increased density in the WT condition. There are a total of 24,690 H3K4me3 peaks, with 15,547 of them located at TSS. Among these, 750 peaks showed decreased H3K4me3 signals in BRG1-depleted cells, with 257 of them situated at TSS. Additionally, there are 95 peaks showing increased H3K4me3 signals in BRG1-depleted cells, with 49 of them located at TSS.

b. The left panel displays heatmaps depicting the BRG1 fold-change between WT control and AID-mediated BRG1-depleted cells. The right panel illustrates the H3K27ac fold-change between WT and BRG1-depleted cells. These heatmaps are centered around enhancers regions (defined by H3K27ac) that co-localized with BRG1 peaks in either WT or AID CD4 T cells. In the color scale, higher fold-change values greater than 1 indicates increased density in the WT condition.
There are a total of 21,347 H3K27ac peaks, with 8,182 of them are enhancer regions. Among these, 806 peaks showed decreased H3K27ac signals in BRG1-depleted cells, with 585 of them

located at enhancer regions. Additionally, there are 90 peaks showing increased H3K27ac signals in BRG1-depleted cells, with 54 of them situated at enhancer regions.

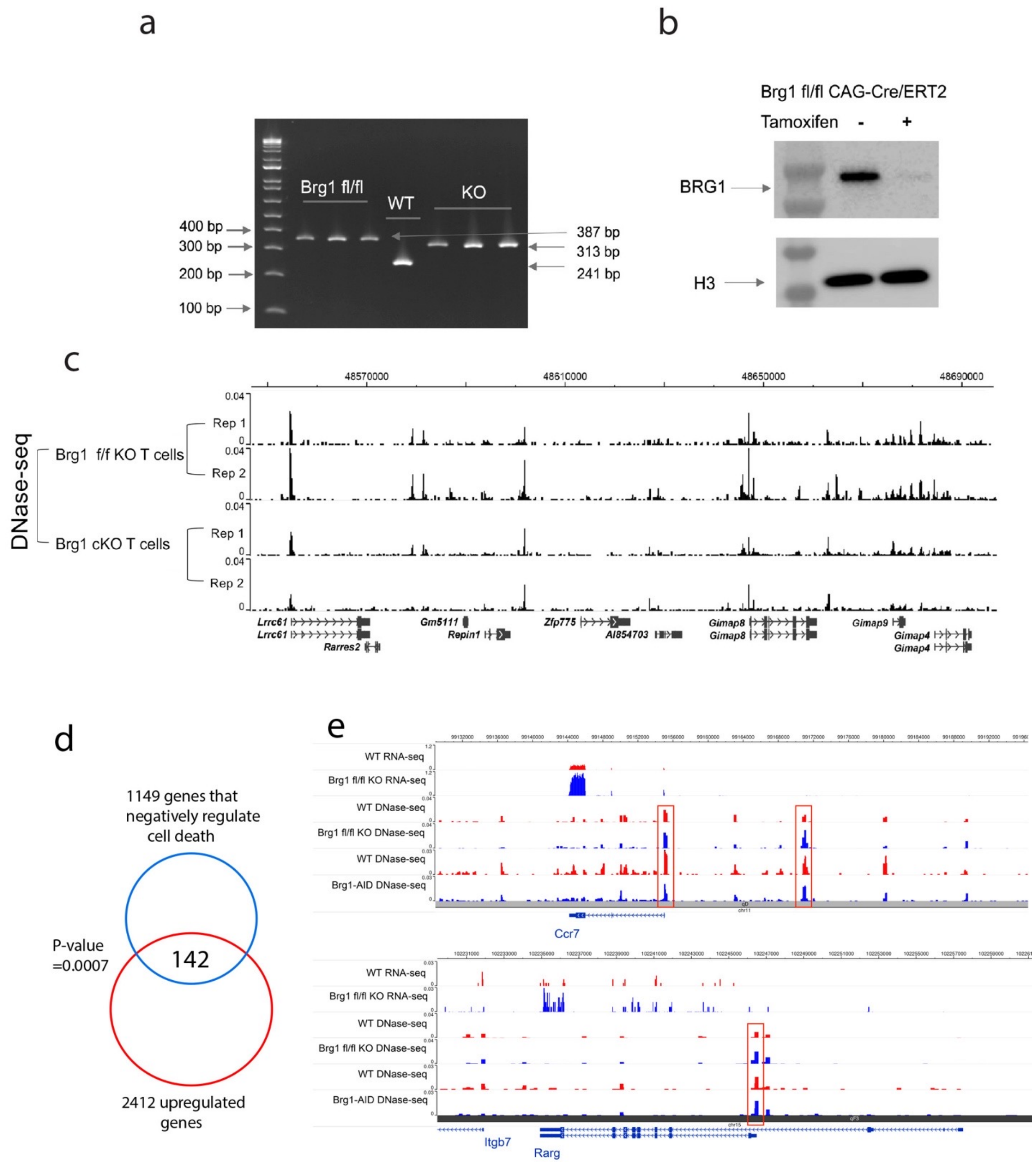

Supplementary Figure 5. *Brg1* fl/fl gene deletion by tamoxifen-induced activation of Cre-ERT in mouse T cells.

a. Genotyping of *Brg1* fl/fl X CAG-Cre/ERT2 and wild type mice. The PCR products for wild type mice, *Brg1* fl/fl, and *Brg1* gene deleted mice are 241bp, 387bp, and 313bp, respectively.

b. Western blotting showing the BRG1 protein levels in *Brg1* fl/fl control cells and *Brg1* gene deleted cells. Histone H3 was used as the loading control.

c. Genome browser snapshot showing the DNase-seq signals in the wild type control T cells (track 1, and 2) and *Brg1* gene deleted T cells (track 3 and 4).

d. A comparison between the genes up-regulated by *Brg1* gene deletion in CD4+ T cells and the genes that negatively regulate cell death. The p-value is calculated by the hypergeometric distribution.

e. Genome Browser images showing RNA-seq data in *Brg1* fl/fl knockout and control CD4+ T cells (top tracks), DNase-seq data in *Brg1* fl/fl knockout and control cells (middle two tracks), and DNase-seq data in AID-mediated BRG1 depletion and control cells (bottom two tracks). The red signals represent wild type control cells and blue track represent *Brg1* f/f knockout cells or AID-mediated depletion cells. Peaks of interest are highlighted by the red rectangles.

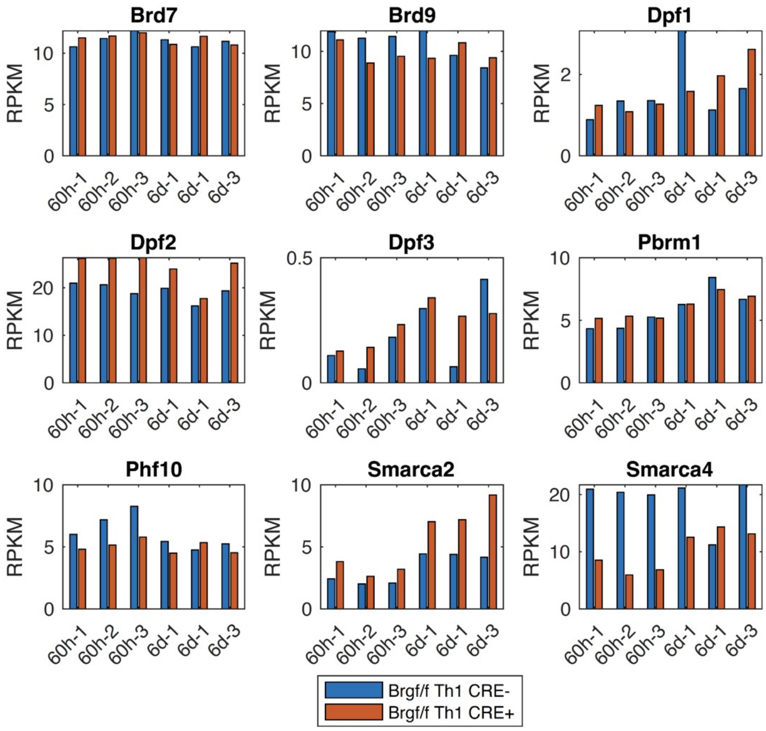

Supplementary Figure 6. Changes of gene expression of established or potential subunits of the SWI/SNF complexes upon *Brg1* f/f gene deletion by CAG-Cre/ERT2 in mouse T cells. The bar plots show the gene expression levels of nine subunits within the SWI/SNF complexes. A comparison is presented between *Brg1* fl/fl CRE- T cells (illustrated by blue bars) and CRE+ T cells (represented by red bars), at both the 60-hour and 6-day time points. Each time point includes three replicates.

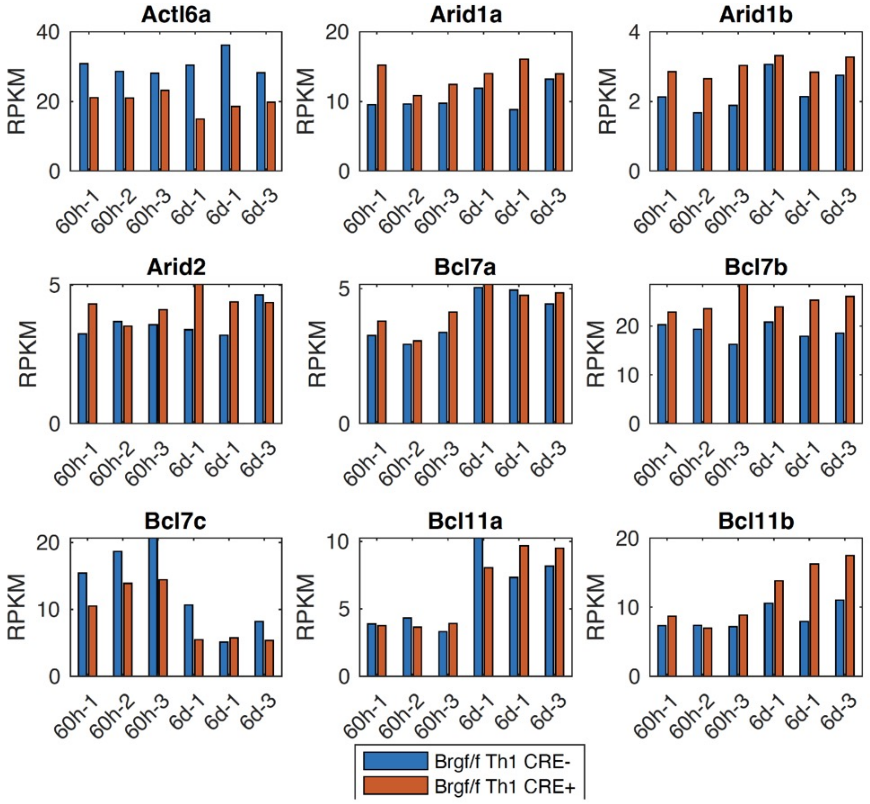

Supplementary Figure 7. Changes of gene expression of established or potential subunits of the SWI/SNF complexes upon *Brg1* f/f gene deletion by CAG-Cre/ERT2 in mouse CD4+ T cells. Similar to Supplementary Figure 6 showing additional nine established or potential subunits within the SWI/SNF complexes.

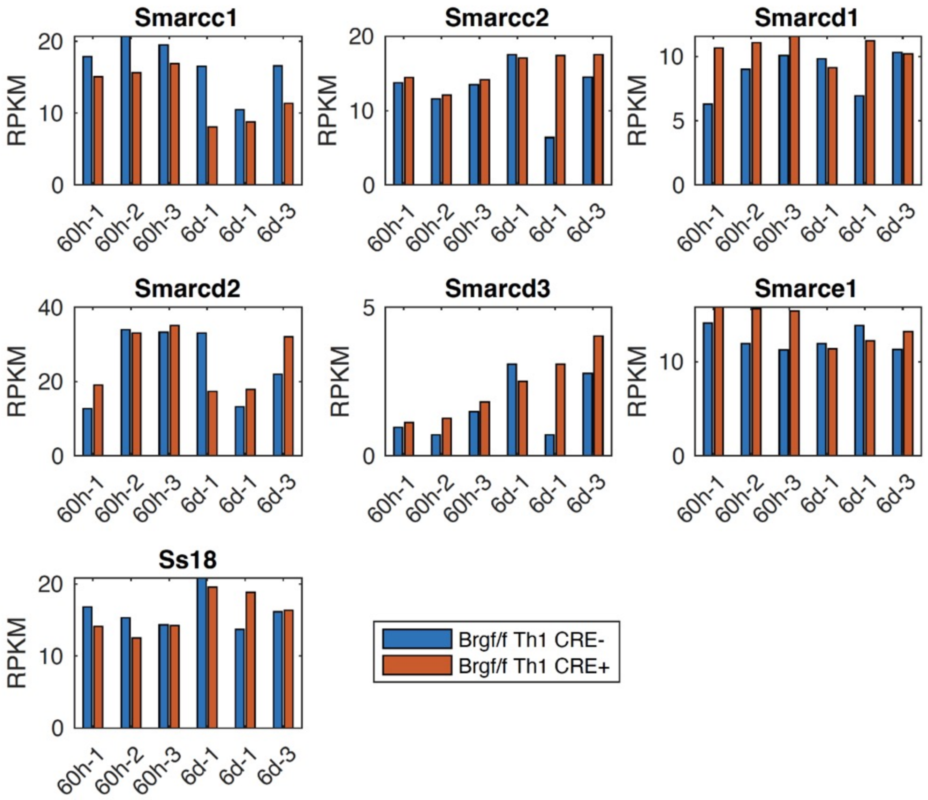

Supplementary Figure 8. Changes of gene expression of established or potential subunits of the SWI/SNF complexes upon *Brg1* f/f gene deletion by CAG-Cre/ERT2 in mouse CD4+ T cells. Similar to Supplementary Fifures 6 and 7, showing additional seven established or potential subunits within the SWI/SNF complexes.

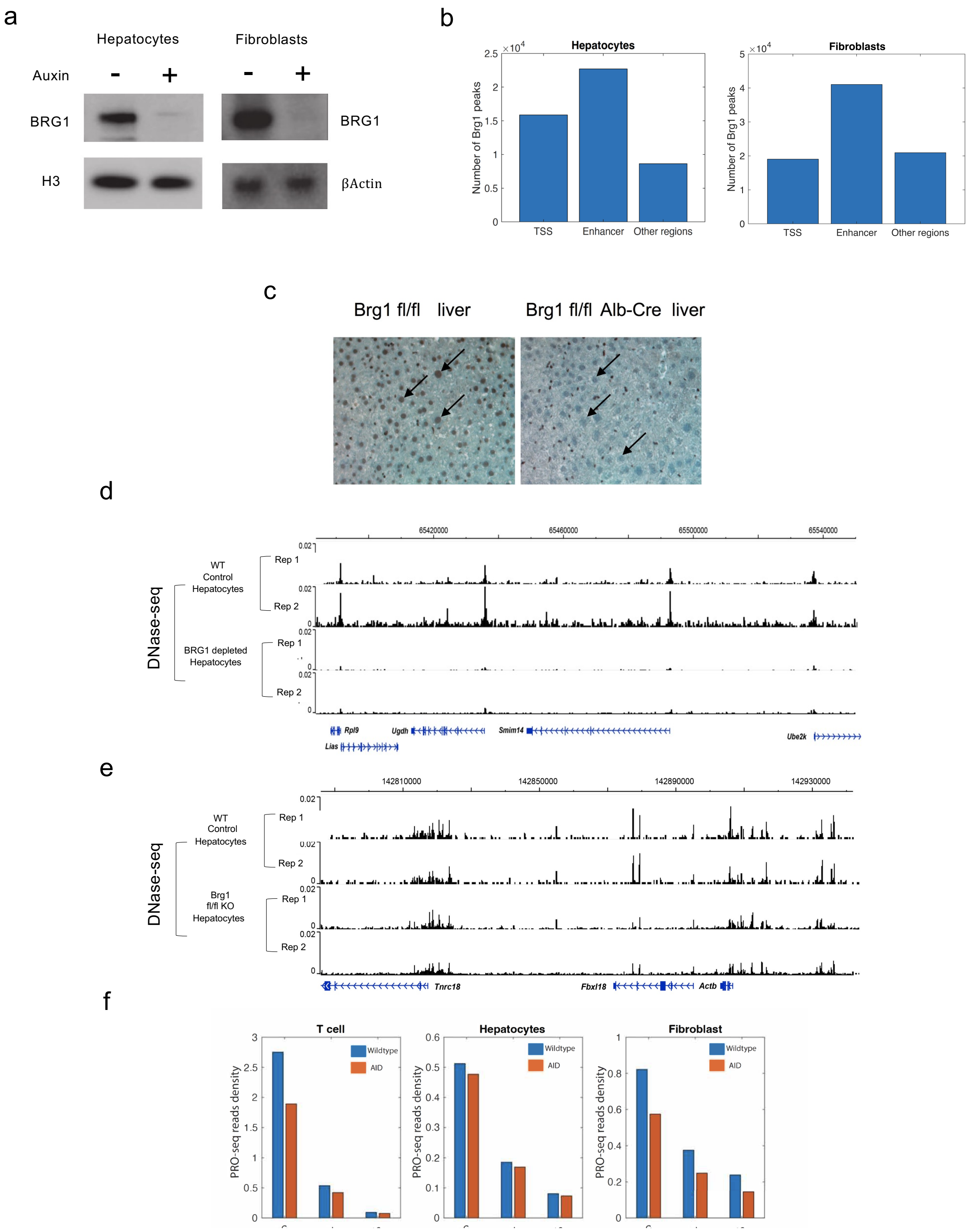

Supplementary Figure 9. BRG1 protein depletion by auxin treatment in hepatocytes and fibroblasts and *Brg1* f/f gene deletion by Alb-Cre in mouse liver.

a. Western blotting showing the BRG1 protein depletion in cultured primary hepatocytes (**left**) and fibroblasts (**right**) by auxin treatment. Primary hepatocytes and fibroblasts isolated from *Brg1-AID* mice were transfected with a Tir1 expression vector for 24 hours and treated with auxin for 10 hours. 50K cells from the BRG1 depleted or wild type control sample were used for Western

blotting. Histone H3 (for hepatocytes) or beta-actin (for fibroblasts) was used as the loading control.

b. Bar plots showing the number of the BRG1 peaks at TSS, enhancers, and other regions in the wild type Hepatocytes (left) and Fibroblasts (right).

c. Immunohistochemistry (IHC) showing the BRG1 protein levels in *Brg1fl/fl* X Alb-Cre and *Brg1fl/fl* mice livers. Livers were collected from adult (12 weeks) male *Brg1*fl/fl X Alb-Cre+ and *Brg*1fl/fl mice, formalin fixed, paraffin embedded, sectioned, and stained with BRG1 antibody (1:1000). The arrows show BRG1-positive staining. Magnification: 40.

d. Genome browser snapshot showing the DNase-seq signals from the wild type control hepatocytes (top tracks 1, 2) and BRG1-AID protein depleted hepatocytes (bottom tracks 3 and 4).

e. Genome browser snapshot showing the DNase-seq signals from the wild type control hepatocytes (top tracks 1, 2) and *Brg1* gene deleted hepatocytes (bottom tracks 3 and 4).

f. Density of PRO-seq data for CD4+ T cells, Hepatocytes, and Fibroblasts at TSS, Gene body, and intergenic regions. Density is the number of reads in the regions multiplied by 10^6 and divided by the region size. The blue bars refer to WT control cells; the red bars refer to BRG1-AID depleted cells by auxin treatment for 10 hours.

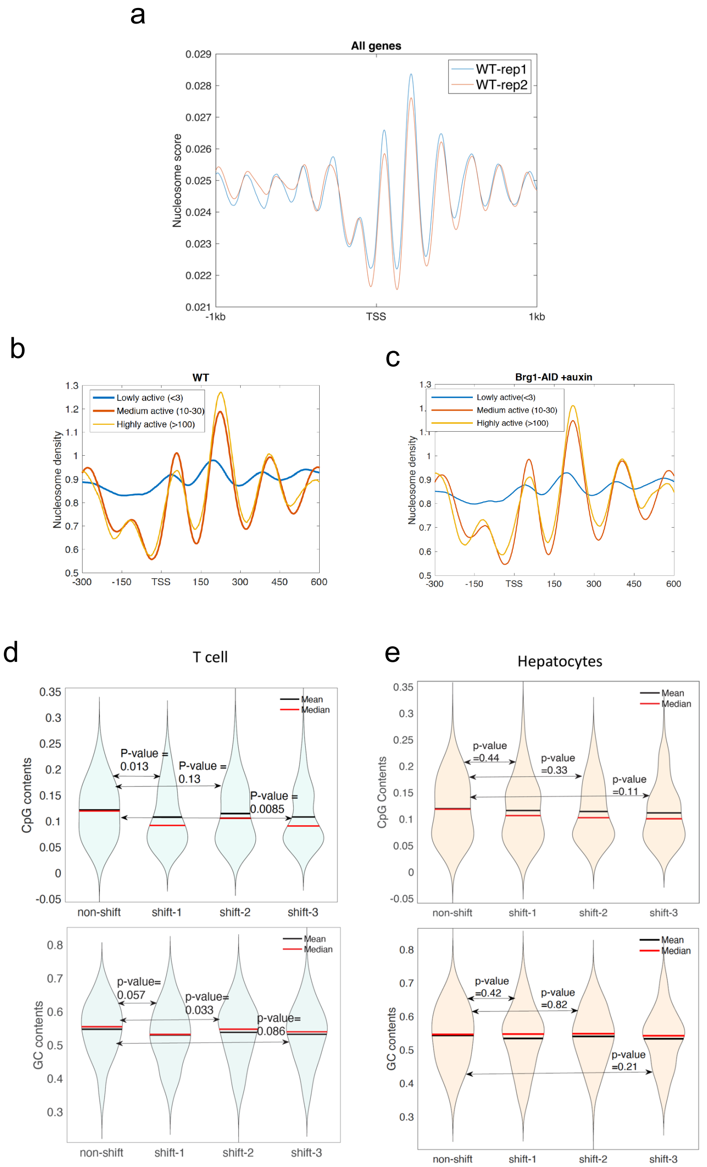

Supplementary Figure 10. MNase-seq revealed similar nucleosome profiles and DNA contents around TSS in wild type (left panel) and BRG1-depleted (right panel) mouse cells.
a. Nucleosome TSS profiles for two replicates of CD4+ T cells in WT mice. CD4+ T cells isolated from *Brg1-AID* mice or wild type control mice were transduced with osTir1 expression retroviral particles for 24 hours and treated with auxin for 10 hours. The cells were then subjected to MNase-seq analysis.
b, c. The MNase-seq profiles surrounding TSSs were plotted for genes with different expression groups: blue line (RPKM <3), orange line (10<RPKM <30), and yellow line (RPKM >100) for wild type control cells (panel **b**) or BRG1-depleted cells (panel **c**).
d. Violin plots showing the CpG contents (**upper panel**) or GC contents (**lower panel**) at different nucleosome shifting patterns in BRG1-depleted CD4+ T cells described in **Fig. 8a**.

e. Violin plots showing the CpG contents (**upper panel**) or GC contents (**lower panel**) at different nucleosome shifting patterns in BRG1-depleted hepatocytes described in **Fig. 8a**.

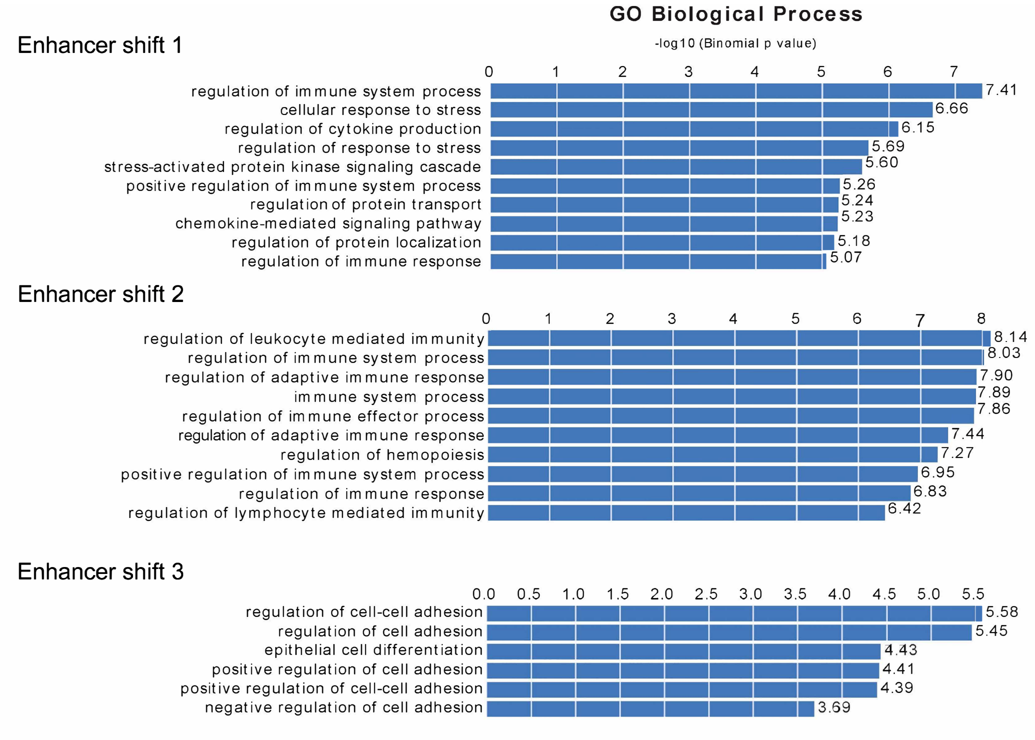

Supplementary Figure 11. GO term enrichment analysis with GREAT for differential nucleosome shifts "shift 1, shift 2, and shift 3" at enhancers.

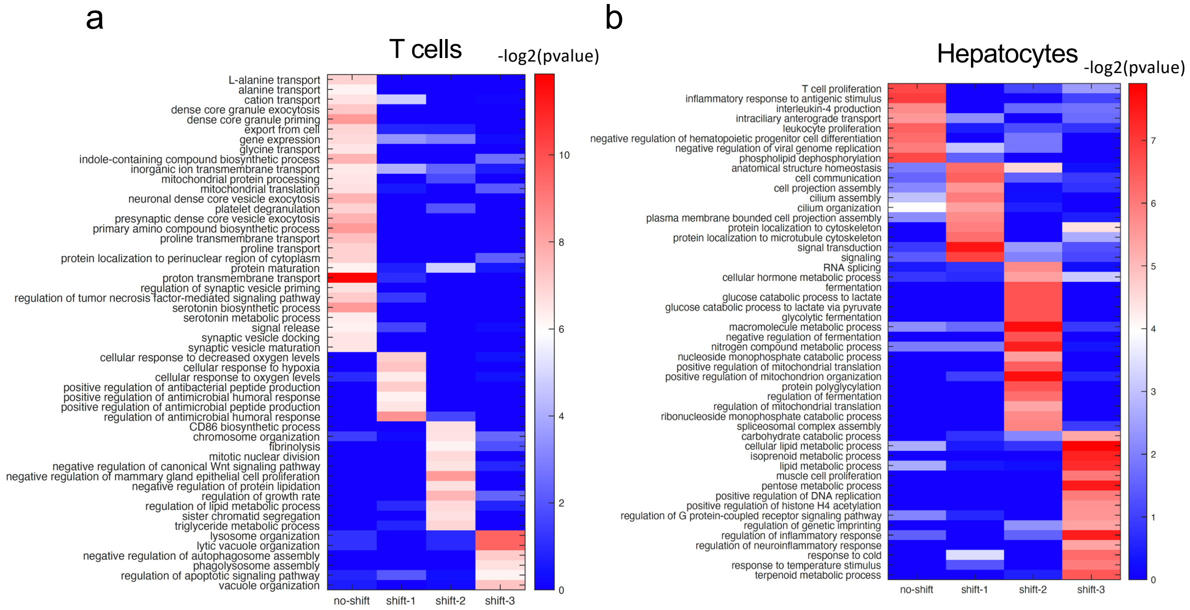

Supplementary Figure 12. Heatmaps for the enrichment of Gene Ontology terms of gene TSSs with different types of nucleosome shifting after acute BRG1 depletion in CD4+ T cells (panel a) and hepatocytes (panel b) as described in Fig. 8a.

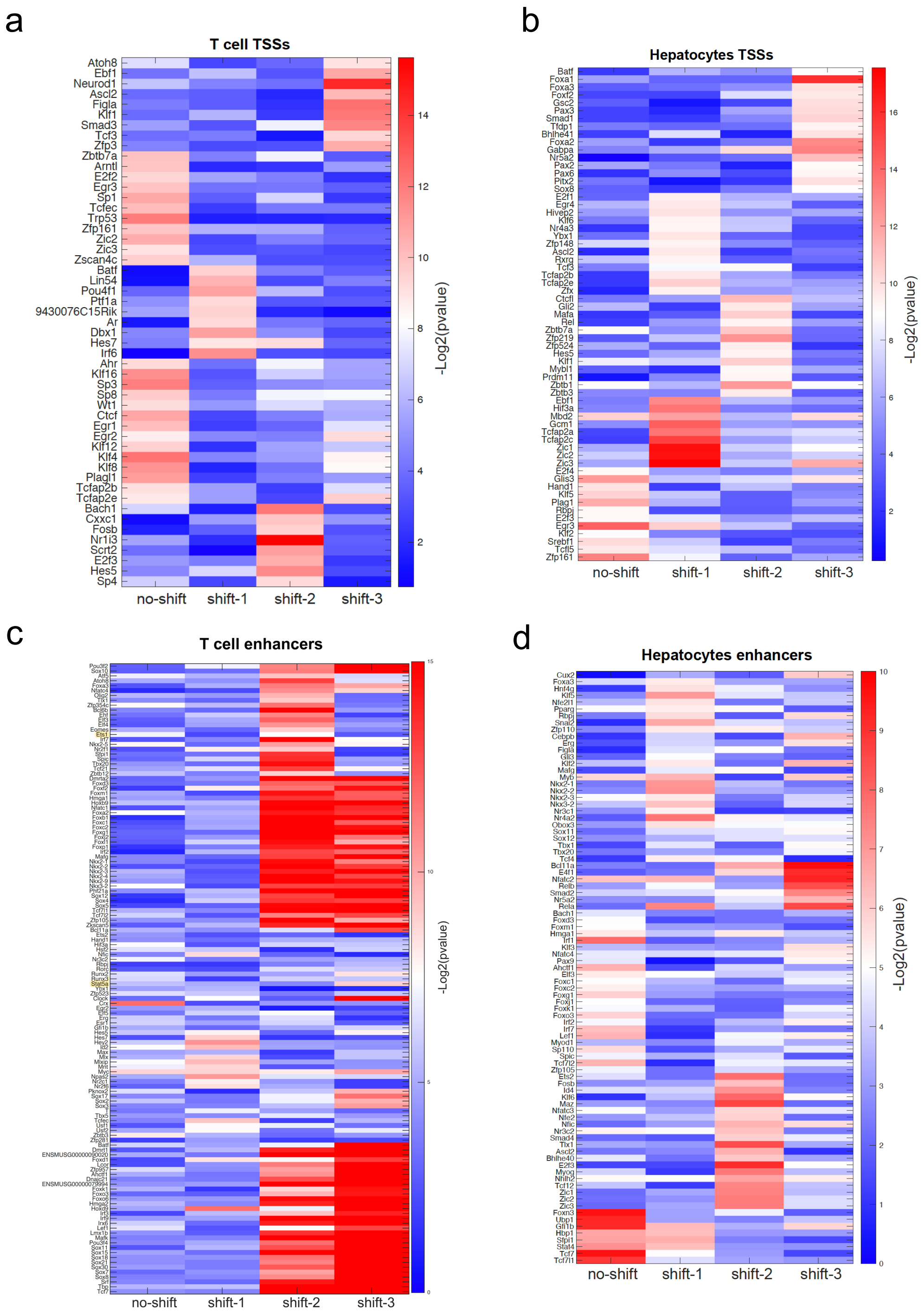

Supplementary Figure 13. Heatmaps for the enrichment of transcription factor binding sites (TFBS) at TSS regions (panel a and b) and enhancer regions (panel c and d) with different types of nucleosome shifting (no-shift, shift-1, shift-2, shift-3).

~~Supplementary Table~~

Studies	Number increased genes (%)	Number of decreased genes (%)	Cells	Experiments	organism
Jesse R. Raab et .al [1]	2662(49%)	2746 (51%)	HepG2 cells	shBrg1	Human
Gangqing Hu et. al. [2]	3077 (55%)	2510 (45%)	CD36+ cells	Brg1 KD	Human
Ana Silvina Nacht et. al. [3]	1334 (77%)	406 (23%)	T47DML cells	siBrg1	Human
Hamish W King et. al. [4]	1226 (47%)	1395 (53%)	ES cells	Brg1 fl/fl conditional KO	Mouse
Gaylor Boulay et. al. [5]	498 (60%)	337 (40%)	SKNMC cells	shRNA KD Brg1	Human
Burak H. Alver et. al. [6]	2377 (71%)	956 (29%)	MEF cells	Brg1 fl/fl conditional KO	Mouse
Joshua Pan et. al. [7]	2009 (56%)	1599 (44%)	BIN-67 cells	ATPase mutant Brg1	Human
Sandra Schick et. al. [8]	1314 (51%)	1260 (49%)	isogenic HAP1 cells	Brg1 KO	Human

Supplementary Table 1. A summary of other Brg1 studies.

Supplementary Reference

1. Raab, J.R., et al., *Co-regulation of transcription by BRG1 and BRM, two mutually exclusive SWI/SNF ATPase subunits.* Epigenetics Chromatin, 2017. **10**(1): p. 62.
2. Hu, G., et al., *Regulation of nucleosome landscape and transcription factor targeting at tissue-specific enhancers by BRG1.* Genome Res, 2011. **21**(10): p. 1650-8.
3. Nacht, A.S., et al., *Hormone-induced repression of genes requires BRG1-mediated H1.2 deposition at target promoters.* EMBO J, 2016. **35**(16): p. 1822-43.
4. King, H.W. and R.J. Klose, *The pioneer factor OCT4 requires the chromatin remodeller BRG1 to support gene regulatory element function in mouse embryonic stem cells.* Elife, 2017. **6**.
5. Boulay, G., et al., *Cancer-Specific Retargeting of BAF Complexes by a Prion-like Domain.* Cell, 2017. **171**(1): p. 163-178.e19.
6. Alver, B.H., et al., *The SWI/SNF chromatin remodelling complex is required for maintenance of lineage specific enhancers.* Nat Commun, 2017. **8**: p. 14648.
7. Pan, J., et al., *The ATPase module of mammalian SWI/SNF family complexes mediates subcomplex identity and catalytic activity-independent genomic targeting.* Nat Genet, 2019. **51**(4): p. 618-626.

8. Schick, S., et al., *Systematic characterization of BAF mutations provides insights into intracomplex synthetic lethalities in human cancers.* Nat Genet, 2019. **51**(9): p. 1399-1410.
