# Supplementary material for: Acute depletion of BRG1 reveals its primary function as an activator of transcription
Source: Nat Commun. 2024 May 29;15:4561. doi: 10.1038/s41467-024-48911-z (PMC11137027; doi:10.1038/s41467-024-48911-z)
Supplement: Supplementary file 3 — Reporting Summary [file 41467_2024_48911_MOESM3_ESM.pdf]

nature portfolio

Corresponding author(s): Keji Zhao

Last updated by author(s): Apr 5, 2024

Reporting Summary

Nature Portfolio wishes to improve the reproducibility of the work that we publish. This form provides structure for consistency and transparency in reporting. For further information on Nature Portfolio policies, see our Editorial Policies and the Editorial Policy Checklist.

Statistics

For all statistical analyses, confirm that the following items are present in the figure legend, table legend, main text, or Methods section.

n/a	Confirmed	
☐	☒	The exact sample size (*n*) for each experimental group/condition, given as a discrete number and unit of measurement
☐	☒	A statement on whether measurements were taken from distinct samples or whether the same sample was measured repeatedly
☐	☒	The statistical test(s) used AND whether they are one- or two-sided<br>*Only common tests should be described solely by name; describe more complex techniques in the Methods section.*
☒	☐	A description of all covariates tested
☐	☒	A description of any assumptions or corrections, such as tests of normality and adjustment for multiple comparisons
☐	☒	A full description of the statistical parameters including central tendency (e.g. means) or other basic estimates (e.g. regression coefficient) AND variation (e.g. standard deviation) or associated estimates of uncertainty (e.g. confidence intervals)
☐	☒	For null hypothesis testing, the test statistic (e.g. *F*, *t*, *r*) with confidence intervals, effect sizes, degrees of freedom and *P* value noted<br>*Give P values as exact values whenever suitable.*
☒	☐	For Bayesian analysis, information on the choice of priors and Markov chain Monte Carlo settings
☒	☐	For hierarchical and complex designs, identification of the appropriate level for tests and full reporting of outcomes
☐	☒	Estimates of effect sizes (e.g. Cohen's *d*, Pearson's *r*), indicating how they were calculated

Our web collection on statistics for biologists contains articles on many of the points above.

Software and code

Policy information about availability of computer code

Data collection	Data collection No software was used for data collection.
Data analysis	Bowtie 2 (v2.2.5) was used for sequencing reads mapping. Ame was used to find enriched motifs. ROTS was used for differential analysis.<br>Other data analysis was performed using custom codes and fully described in Methods.<br>Custom codes for generating figures are available at https://doi.org/10.6084/m9.figshare.24715956.v1.

For manuscripts utilizing custom algorithms or software that are central to the research but not yet described in published literature, software must be made available to editors and reviewers. We strongly encourage code deposition in a community repository (e.g. GitHub). See the Nature Portfolio guidelines for submitting code & software for further information.

Data

Policy information about availability of data

All manuscripts must include a data availability statement. This statement should provide the following information, where applicable:

- Accession codes, unique identifiers, or web links for publicly available datasets
- A description of any restrictions on data availability
- For clinical datasets or third party data, please ensure that the statement adheres to our policy

The DNase-seq, MNase-seq, ChIC-seq, PRO-seq and RNA-seq data generated in this study have been deposited in the GEO database under accession code

GSE156569 (https://www.ncbi.nlm.nih.gov/geo/query/acc.cgi?acc=GSE156569). The processed data of figures are available at FigShare [https://doi.org/10.6084/m9.figshare.24578932.v1].

Research involving human participants, their data, or biological material

Policy information about studies with human participants or human data. See also policy information about sex, gender (identity/presentation), and sexual orientation and race, ethnicity and racism.

Reporting on sex and gender	This information is not collected
Reporting on race, ethnicity, or other socially relevant groupings	No applicable
Population characteristics	No applicable
Recruitment	No applicable
Ethics oversight	No applicable

Note that full information on the approval of the study protocol must also be provided in the manuscript.

Field-specific reporting

Please select the one below that is the best fit for your research. If you are not sure, read the appropriate sections before making your selection.

☒ Life sciences ☐ Behavioural & social sciences ☐ Ecological, evolutionary & environmental sciences

For a reference copy of the document with all sections, see nature.com/documents/nr-reporting-summary-flat.pdf

Life sciences study design

All studies must disclose on these points even when the disclosure is negative.

Sample size	Sample size All studies have at least two replicates since most softwares for calculation the differential differences required at least two replicates
Data exclusions	No data is excluded
Replication	All experiments have at least two replicates.
Randomization	N/A
Blinding	This study is in vitro experiment and no blinding is required. The investigators were not blinded during data collection and analysis.

Reporting for specific materials, systems and methods

We require information from authors about some types of materials, experimental systems and methods used in many studies. Here, indicate whether each material, system or method listed is relevant to your study. If you are not sure if a list item applies to your research, read the appropriate section before selecting a response.

Materials & experimental systems

n/a	Involved in the study	
☐	☒	Antibodies
☐	☒	Eukaryotic cell lines
☒	☐	Palaeontology and archaeology
☐	☒	Animals and other organisms
☒	☐	Clinical data
☒	☐	Dual use research of concern
☒	☐	Plants

Methods

n/a	Involved in the study	
☒	☐	ChIP-seq
☒	☐	Flow cytometry
☒	☐	MRI-based neuroimaging

Antibodies

Antibodies used	1ul antibodies (anti-H3K4me3 (17-614, Millipore), anti-H3K27ac (ab4729, Abcam), anti-p300 (sc-584, Santa Cruz), anti-BRG1 (ab110641, Abcam), anti-RNA Polymerase II (ab817, Abcam)) and 3ul protein A-MNase fusion protein were mixed well and incubated at 4°C for 30 minutes. Next, the antibody-pA-MNase complex was added to the 100ul permeabilized.<br><br>Western blot analysis<br>300,000 CD4+ T cells, hepatocytes and 40,000 fibroblasts lysed in 50 µl 2 X SDS loading buffer with 10% DTT were resolved by the Novex NuPage SDS-PAGE gel system (Life Technologies). Proteins were transferred to Supported Nitrocellulose Membrane (Bio-Rad) and were incubated with anti-Brg1 (sc-10768, Santa Cruz, 1:1000), anti-osTIR1(PD048, MBL life science, 1:1000), and anti-GAPDH (clone: AC-15, Sigma-Aldrich, 1:5000), anti-panH3 (ab21054, Abcam, 1:5000) and anti-actin (A2228, Sigma, 1:5000). Blots were visualized with Pierce ECL Western Blotting Substrate (Thermo Scientific).
Validation	The qPCR is performed before library preparation.

Eukaryotic cell lines

Policy information about cell lines and Sex and Gender in Research

Cell line source(s)	HEK293Ts (a human embryonic kidney female cell line), ATCC, CRL-3216
Authentication	HEK293Ts were authenticated by visual inspection
Mycoplasma contamination	Mycoplasma tested negative
Commonly misidentified lines<br>(See ICLAC register)	NO

Animals and other research organisms

Policy information about studies involving animals; ARRIVE guidelines recommended for reporting animal research, and Sex and Gender in Research

Laboratory animals	3~5 6-10 weeks old mice were used for each experiment with C57BL/6 background.
Wild animals	No wild animals were used in this study
Reporting on sex	The mice sex and gender were not considered in this study because the gender didn't affect the results.
Field-collected samples	No field-collected samples in this study
Ethics oversight	This study was approved by the National Institute of Health and complied with all relevant ethical regulations with the protocol number H-0141R2

Note that full information on the approval of the study protocol must also be provided in the manuscript.

Plants

Seed stocks	*Report on the source of all seed stocks or other plant material used. If applicable, state the seed stock centre and catalogue number. If plant specimens were collected from the field, describe the collection location, date and sampling procedures.*
Novel plant genotypes	*Describe the methods by which all novel plant genotypes were produced. This includes those generated by transgenic approaches, gene editing, chemical/radiation-based mutagenesis and hybridization. For transgenic lines, describe the transformation method, the number of independent lines analyzed and the generation upon which experiments were performed. For gene-edited lines, describe the editor used, the endogenous sequence targeted for editing, the targeting guide RNA sequence (if applicable) and how the editor was applied.*
Authentication	*Describe any authentication procedures for each seed stock used or novel genotype generated. Describe any experiments used to assess the effect of a mutation and, where applicable, how potential secondary effects (e.g. second site T-DNA insertions, mosiacism, off-target gene editing) were examined.*
